# Comparison of bone marrow and adipose tissue-derived canine mesenchymal stem cells

**DOI:** 10.1186/1746-6148-8-150

**Published:** 2012-08-31

**Authors:** Hiroshi Takemitsu, Dongwei Zhao, Ichiro Yamamoto, Yasuji Harada, Masaki Michishita, Toshiro Arai

**Affiliations:** 1 Department of Veterinary Science, School of Veterinary medicine, Nippon Veterinary and Life Science University, 1-7-1 Kyonancho, Musashino, Tokyo, 180-8602, Japan; 2 Veterinary medical teaching hospital, Nippon Veterinary and Life Science University, 1-7-1 Kyonan-cho, Musashino-shi, Tokyo, 180-8602, Japan; 3 Department of Veterinary Pathology, Nippon Veterinary and Life Science University, 1-7-1 Kyonan-cho, Musashino, Tokyo, 180-8602, Japan

**Keywords:** Canine, Mesenchymal stem cell, Cell surface markers, Embryonic stem cell markers

## Abstract

**Background:**

Bone marrow-derived mesenchymal stem cells (BM-MSCs) and adipose tissue-derived mesenchymal stem cells (AT-MSCs) are potential cellular sources of therapeutic stem cells. MSCs are a multipotent population of cells capable of differentiating into a number of mesodermal lineages. Treatment using MSCs appears to be a helpful approach for structural restoration in regenerative medicine. Correct identification of these cells is necessary, but there is inadequate information on the MSC profile of cell surface markers and mRNA expression in dogs. In this study, we performed molecular characterization of canine BM-MSCs and AT-MSCs using immunological and mRNA expression analysis.

**Results:**

Samples were confirmed to be multipotent based on their osteogenic and adipogenic differentiation. And these cells were checked as stem cell, hematopoietic and embryonic stem cell (ESC) markers by flow cytometry. BM- and AT-MSCs showed high expression of CD29 and CD44, moderate expression of CD90, and were negative for CD34, CD45, SSEA-3, SSEA-4, TRA-1-60, and TRA-1-81. SSEA-1 was expressed at very low levels in AT-MSCs. Quantitative real-time PCR (qRT-PCR) revealed expression of Oct3/4, Sox2, and Nanog in BM- and AT-MSCs. There was no significant difference in expression of Oct3/4 and Sox2 between BM-MSCs and AT-MSCs. However, Nanog expression was 2.5-fold higher in AT-MSCs than in BM-MSCs. Using immunocytochemical analysis, Oct3/4 and Sox2 proteins were observed in BM- and AT-MSCs.

**Conclusion:**

Our results provide fundamental information to enable for more reproducible and reliable quality control in the identification of canine BM-MSCs and AT-MSCs by protein and mRNA expression analysis.

## Background

Mesenchymal stem cells (MSCs) have been successfully isolated from bone marrow [1] and adipose tissue [2,3] in humans. MSCs are multipotent and can differentiate not only into cells of the mesodermal lineage, such as osteoblasts [4], chondrocytes [5], and adipocytes [6], but also into neurocytes [7] and cardiomyocytes [8]. Given the appropriate microenvironment, MSCs can differentiate into various tissues. Due to their accessibility, expandability, and multipotentiality, MSCs hold promise for applications in regenerative medicine [9,10].

MSCs are defined by their plastic adherent growth and subsequent expansion under specific culture conditions and by their *in vitro* and *in vivo* differentiation potential [1-8]. Induction of differentiation into osteoblasts and adipocytes under appropriate culture conditions has been extensively demonstrated [11]. However, MSC cultures are composed of heterogeneous cell populations. The proportion of pluripotent stem cells in bone marrow-derived whole cell cultures ranged from 1/10,000 to 1/100,000 [12,13]. The lack of common standards and precise definition of initial cell preparations remains a major obstacle in research on MSCs and their application. Current research aims to characterize MSCs and to find ways of expanding MSC cultures and maintaining the cells in the undifferentiated state [14-20].

The expression profile of cell surface markers and mRNAs is well characterized in other species. In many studies in humans and dogs CD29, CD44 and CD90 were regarded as positive cell-surface markers for MSCs [14-17,21], while CD34 and CD45 were regard as negative surface markers [18-20,22]. In addition, stage-specific embryonic antigen (SSEA)-1, SSEA-3, SSEA-4; the keratin sulfate-associated antigen tumor-related antigen (TRA)-1-60, and TRA-1-81 were reported as markers of canine embryonic stem cells (ESCs) [23]. These molecules constitute a comprehensive set of unique stem cell markers. Moreover, Oct3/4, Sox2, and Nanog were shown to be important transcription factors regulating ESC self-renewal and differentiation [24,25]. These transcription factors interact with each other to oversee a vast regulatory network that maintains pluripotency and inhibited differentiation [24].

In veterinary medicine, the use of MSCs for tissue repair is helpful and is likely to increase in future. However, there have been few studies on cell surface markers and mRNA expression profiles of canine MSCs. Here we evaluated the canine BM- and AT-MSC cell surface markers CD29, CD44, CD90, CD34, CD45, SSEA-1, SSEA-3, SSEA-4, TRA-1-60, and TRA-1-81 by flow cytometry. We also analyzed the mRNA expression profile of Oct3/4, Sox2, and Nanog in canine BM- and AT-MSCs by quantitative real-time PCR (qRT-PCR). Finally, we used immunocytochemistry to examine the expression and localization of Oct3/4 and Sox2. The aim of this study was the biological characterization of canine MSCs isolated from bone marrow and adipose tissue.

## Results

### Cell isolation and culture

**Figure 1:**
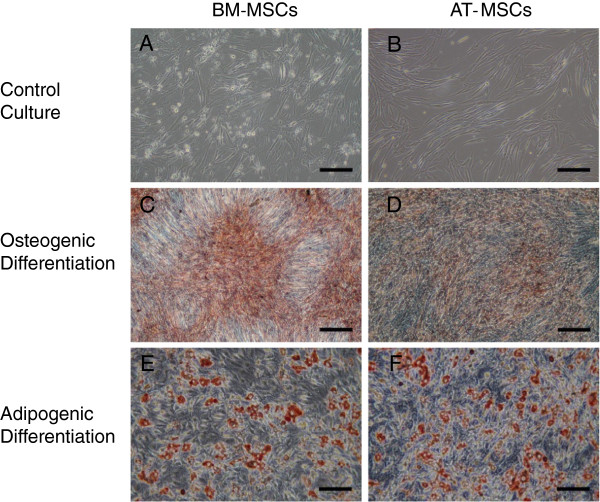
**In vitro differentiation of BM-MSCs and AT-MSCs.** Both types of cell were maintained in control medium** (A, B). **Osteogenic differentiation was identified by von Kossa staining** (C, D) **and adipogenic differentiation by Oil Red O staining** (E, F).** Scale bar, 200 μm.

Adherent cells were observed at the bottom of the culture flasks within 2 days; these cells were isolated from both bone marrow and adipose tissue after plating. Bone marrow-derived cells formed several colonies and proliferated, taking various shapes, including discoidal flat, triangular, and elongated (Figure1A). Bone marrow-derived cells were uniformly distributed immediately after passage, but thereafter, gradually formed colonies and proliferated. Unlike bone marrow-derived cells, adipose-derived cells were uniformly distributed and no colony formation was observed (Figure1B).

### In vitro differentiation

Osteogenic differentiation was demonstrated histochemically using von Kossa stain (Figure1C, D). After adipogenic induction culture for 3 weeks, MSCs showed an adipogenic phenotype. Histochemical staining with Oil Red O was used to demonstrate adipogenic differentiation of MSCs. Lipid droplets were observed at about 14 days of culture in adipogenic medium and were positive for Oil Red O staining (Figure1 E, F). Both differentiation experiments were carried out in passage 2 (Figure1C- F).

### Characterization of surface markers for MSC

**Figure 2:**
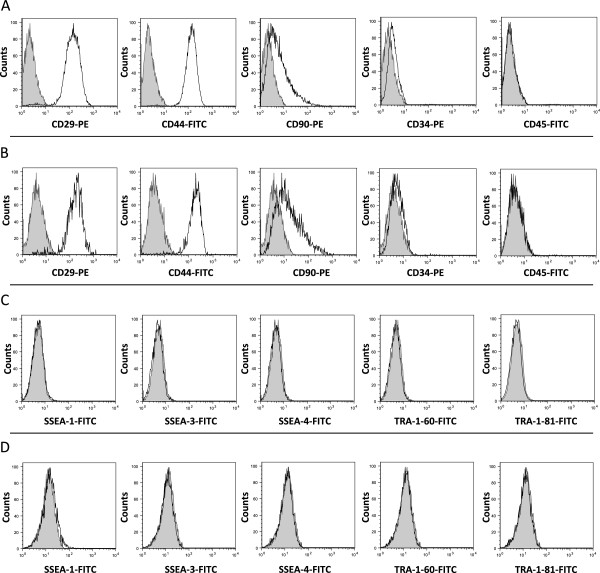
**Flow cytometry.** Comparison of cell surface proteins CD29, CD44, CD90, CD34, CD45, SSEA-1, SSEA-3, SSEA-4, TRA-1-60, and TRA-1-81 on primary cultures of BM-MSCs **(A, C)** and AT-MSCs **(B, D).** Solid histograms show nonspecific staining and open histograms show specific staining for the indicated marker. Three different donor MSC populations from each tissue type were analyzed and representative samples are shown.

Cell surface antigen phenotyping was performed on BM- and AT-MSCs by flow cytometry (Figure2) (Table1). BM-MSCs and AT-MSCs revealed very similar expression patterns of surface markers. Adhesion molecule protein CD29 (BM-MSCs, 98.41 ± 0.53%; AT-MSCs, 97.85 ± 0.94%), receptor molecule protein CD44 (BM-MSCs, 98.90 ± 0.25%; AT-MSCs, 97.85 ± 0.85%), and thy-1 CD90 (BM-MSCs, 19.10 ± 2.1%; AT-MSCs, 22.55 ± 2.8%) were expressed in both BM-MSCs (Figure2A) and AT-MSCs (Figure2B). Both types of cells also expressed CD73 (BM-MSCs, 0.0081 ± 0.0081%; AT-MSCs, 0.038 ± 0.038%), CD105 (BM-MSCs, 0.104 ± 0.03%; AT-MSCs, 0.023 ± 0.018%) (data not shown).

**Table 1:** Summary of cell surface markers on canine BM- and AT-MSCs

	**BM-MSCs**	**AT-MSCs**
Mesenchymal Stromal Cell Marker		
CD29	**++**	**++**
CD44	++	++
CD90	+	+
Hematopoietic markers		
CD34	-	-
CD45	-	-
Embryonic stem cell markers		
SSEA-1	-	**-**
SSEA-3	-	-
SSEA-4	-	-
TRA-1-60	-	-
TRA-1-81	-	-

Canine MSCs were analyzed by FACS, as shown in Figure2. Samples were scored as “−” if <9% of cells were positive, “+” if 9%–69% were positive, and “++” if ≥70% were positive.

Hematopoietic markers CD34 (BM-MSCs, 0.88 ± 0.21%; AT-MSCs, 0.25 ± 0.06%) and CD45 (BM-MSCs, 0.24 ± 0.07; AT-MSCs, 0.17 ± 0.02) were detected in BM-MSCs (Figure2A) and AT-MSCs (Figure2B).

BM-MSCs (Figure2C) and AT-MSCs (Figure2D) expressed the embryonic stem cell-specific markers SSEA-1 (BM-MSCs, 0.12 ± 0.03%; AT-MSCs, 1.40 ± 0.11%), SSEA-3 (BM-MSCs, 0.00 ± 0.00%; AT-MSCs, 0.01 ± 0.01%), SSEA-4 (BM-MSCs, 0.07 ± 0.01%; AT-MSCs, 0.02 ± 0.01%), TRA-1-60 (BM-MSCs, 0.01 ± 0.00%; AT-MSCs, 0.02 ± 0.01%), and TRA-1-81 (BM-MSCs, 0.00 ± 0.00%; AT-MSCs, 0.01 ± 0.01%).

### mRNA expression analysis using quantitative real-time PCR

**Figure 3:**
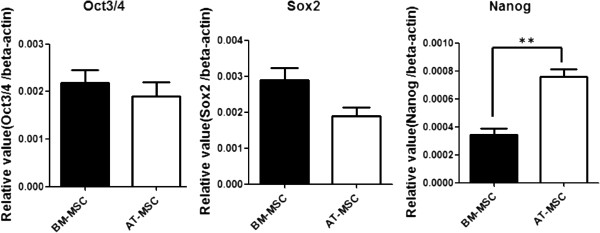
**Quantitative RT-PCR.** Expression levels of mRNAs for stem cell markers Oct3/4, Sox2, and Nanog in BM-MSCs and AT-MSCs. Each value was normalized to beta-actin expression. Statistical comparisons were made using Student’s *t* test (**p < 0.01).

Expression levels of canine Oct3/4, Sox2, and Nanog mRNA in BM- and AT-MSCs were examined by qRT-PCR (Figure3). Expression of Oct3/4 did not differ significantly difference between BM-MSCs and AT-MSCs. However, Sox2 expression tended to be higher in BM-MSCs than in AT-MSCs while Nanog expression in AT-MSCs was 2.5-fold higher than in BM-MSCs. (*p* < 0.01).

### Immunocytochemistry

**Figure 4:**
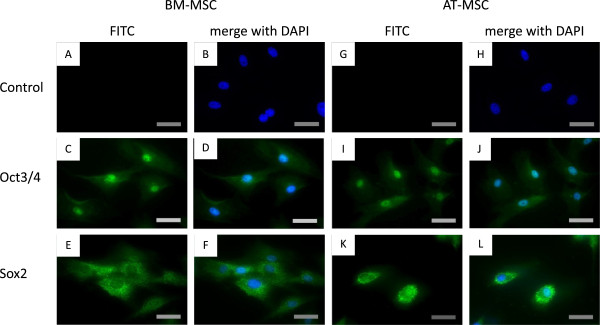
**Immunocytochemistry.** Expression and localization of Oct3/4 and Sox2 in BM-MSCs and AT-MSCs. Immunofluorescent localization of Oct3/4 in BM-MSCs with DAPI counterstaining **(C, D).** Immunofluorescent localization of Sox2 in BM-MSCs with DAPI counterstaining** (E, F).** Immunofluorescent localization of Oct3/4 in AT-MSCs with DAPI counterstaining **(I, J).** Immunofluorescent localization of Oct3/4 in BM-MSCs with DAPI counterstaining** (K, L). **Scale bar, 40 μm.

Immunocytochemistry was used to analyze Oct3/4 and Sox2 proteins (Figure4). In both BM-MSCs and AT-MSCs, Oct3/4 was detected mainly the nuclei, whereas Sox2 was detected in the cytoplasm.

## Discussion

In this study, we confirmed the potential of MSCs to differentiate into osteoblasts or adipocytes, and evaluated the protein and mRNA expression profiles of these cells. In addition, we compared the expression patterns of ESC markers and germ layer markers in MSCs derived from bone marrow and adipose tissue. BM-MSC and AT-MSC populations expressed CD29, CD44, and CD90 with similar intensity. In particular, in both BM- and AT-MSCs, more than 95% of the cell population expressed CD29 and CD44. Our results indicate that expression of CD90 was lower than that of other markers. However, many human studies have reported strong expression of CD90. This may be related to differences within species. Many human studies have reported positive expression of CD90 whereas many studies in mice have reported negative expression of CD90 [11,26]. In addition, the decline in CD90 expression may be influenced by the passage number of the cells. In early passage cells, CD90 showed variable expression [25,27-32], and Riekstina et al. reported that CD90 expression decreased with increasing passage number [33]. CD34 and CD45 markers are usually associated with hematopoietic stem cells.

The qRT-PCR analysis revealed expression of the stem cell markers Oct3/4, Sox2, and Nanog in canine BM- and AT-MSCs. In a human study, mRNAs of these markers were expressed in human MSCs [34]. These transcription factors mediate self-renewal and cell-fate specification and are downregulated when the cells are completely differentiated [35]. Our results reflect their undifferentiated phenotype and high propensity for pluripotent differentiation and support the hypothesis that MSCs may be pluripotent stem cells deposited in tissues during development. Oct3/4 mRNA showed similar levels of expression in BM- and AT-MSCs. However, expression of Sox2 in BM-MSCs tended to be higher than in AT-MSCs. Sox2 belongs to the Sox subfamily, whose members are defined by the relationship of their HMG box [36]. In addition, Sox2 regulates Oct3/4 expression and maintains ESC pluripotency through upstream transcription factors [37] and through cooperative binding of OCT3/4 to DNA [38]. However, our results indicate Oct3/4 expression was similar in BM- and AT-MSCs, but Sox2 expression slightly differed between the two cell types. This is probably due to the additional expression of other Sox family members that act in a compensatory in MSCs [37]. In addition, expression of Nanog in AT-MSCs was 2.5-fold higher than in BM-MSCs. Nanog is required to maintain the undifferentiated state and for the self-renewal of stem cells. In ESCs, knockout or knockdown of Nanog abolishes both self-renewal and pluripotency, and results in differentiation of extraembryonic endoderm [39,40]. In a human study, comparison of proliferation in BM- and AT-MSCs revealed high proliferative ability in AT-MSCs [41]. These results suggest that AT-MSCs may maintain the undifferentiated state and that their self-renewal ability is greater than that of BM-MSCs.

We performed immunostaining with Oct3/4 and Sox2 in MSCs. In both BM- and AT-MSCs, Oct3/4 was detected in the nuclear fraction, whereas Sox2 was detected in the cytoplasmic fraction. Several transcription factors are known to be localized in the cytoplasmic fraction, such as The Tead [42] and FOXO families [43]. These studies showed inhibitory activity of the transcription factors. Hence it is possible that they inactivate Sox2. In addition, Sox2 was localized in the nucleus in canine ESCs [23]. There are many functional differences between ESCs and MSCs. For example, ESCs form teratomas in the testis, but MSCs do not. We therefore suggest that restricted localization of Sox2 protein may lead to lack of proliferation of MSCs *in vivo* as well as maintenance of pluripotency of MSCs *in vitro*.

## Conclusion

Our study reveals the protein and mRNA expression profiles of canine BM- and AT-MSCs. The two types of cells showed similar cell surface marker profiles. Quantitative real-time PCR revealed expression of mRNAs for Oct3/4, Sox2, and Nanog in BM- and AT-MSCs. The localization of Oct3/4 and Sox2 was demonstrated immunocytochemically. mRNA expression of Nanog was greater in AT-MSCs than in BM-MSCs. Further studies are required to characterize canine MSCs with respect to the expression of other proteins and mRNAs.

## Methods

### MSC isolation and culture

Four young healthy female beagle dogs (1 year old, 9.5-11.3 kg body weight) were used. All animals were anesthetized with propofor (Hospira, Osaka, Japan) (7 mg/kg by intravenous injection) before tissue sample were taken. After incubation, anesthesia was maintained with isoflurane (1.5–2.0%) in oxygen. Animal experiments were carried out in accordance with the National Institutes of Health guidelines for the care and use of laboratory animals. The protocol of this study was approved by the University Committee for Animal Experimentation.

### BM-MSCs

Under general anesthesia, bone marrow was aspirated from the proximal humerus using a general bone marrow biopsy technique. Briefly, a sterilized 13-gauge Jamshidi needle (Cardinal Health, McGaw Park, USA) was used to aspirate 5 ml of bone marrow into a syringe containing 5 ml of heparinized (1,000 units/ml) saline solution. Perioperative analgesic management was carried out by the pre- and post-operative administration of buprenorphine, which was administered twice daily for 3 consecutive days after surgery. In addition, ampicillin (25 mg/kg) was orally administered twice daily for up to 7 days after surgery.

The bone marrow collected was dissociated and then resuspended with a pipette. The suspension was centrifuged for 5 min at 300 × *g* and collected as a pellet. Marrow cells were then resuspended in 10 ml of 10% FBS–PBS (FBS, Invitrogen, Carlsbad, USA; PBS, Invitrogen). To obtain MSC-enriched nucleated cells, density separation (1.077 g/ml) was performed using Lymphoprep (Axis-Shield, Oslo, Norway). A suspension of marrow cells in FBS–PBS solution (10 ml) was carefully layered onto 5 ml of Lymphoprep. Separation was achieved by centrifugation at 800 × *g* for 30 min at room temperature. The nucleated cells collected from the PBS solution - Lymphoprep interface were then washed in PBS and transferred into T-75 cell culture flasks with 10 ml of control medium consisting of Dulbecco’s modified Eagle’s medium (Invitrogen), 10% FBS, and 1% antibiotic–antimycotic solution. Cells were plated at a density of 1–5 × 10^7^ cells/plate and incubated at 37°C in a humidified 5% CO_2_ incubator and the medium was changed twice weekly. When primary cultures reached 70%–80% confluency, attached cells were passaged by exposure to 0.25% trypsin, 1 mM EDTA (Invitrogen) for 3 min, and replated at a density of 8.0 × 10^3^ cells/cm^2^ for subsequent passage.

### AT-MSCs

Adipose tissues were also harvested from each dog under general anesthesia. Subcutaneous fat pads (approximately 1.0 g) were harvested from the inguinal area. These pads were finely minced with scissors and then digested in 40 ml of PBS containing 0.15% collagenase (Invitrogen), with vigorous shaking for 60 min at 37°C. Samples were then filtered using 100 μm cell strainers (BD Biosciences, Franklin Lakes, USA) and washed with PBS. The cells obtained were seeded into T-75 cell culture flasks with 10 ml of control medium and incubated in the same manner as bone marrow cells.

### In vitro differentiation

For osteogenic differentiation, passage 2 BM-MSCs and AT-MSCs were plated on 6-well culture plates at a density of 5.0 × 10^3^ cells/cm^2^, and after incubation in control medium for 24 h, the medium was changed to osteogenic medium. The osteogenic medium was Canine Osteoblast Differentiation Medium purchased from Cell Applications (San Diego, USA). The medium was changed twice weekly. For osteogenic analysis, mineral deposits were quantitatively analyzed by von Kossa (Sigma, St. Louis, USA) staining at 14 days.

For adipogenic differentiation, passage 2 BM- and AT-MSCs were plated on 6-well culture plates at a density of 8.0 × 10^3^ cells/cm^2^. The cells were cultured in control medium until confluency, and then the medium was changed to Canine Adipocyte Differentiation Medium (Cell Applications). The medium was changed twice in a week. Oil Red O (Sigma) staining was performed to analyze adipogenesis at 14 days.

### Flow cytometry

Passage 2 BM-MSCs and AT-MSCs were placed in FACS tubes (BD Biosciences) at 2 × 10^5^ cells/tube, washed with FACS buffer (PBS containing 1% sodium azide and 1% FBS, pH 7.2). The cells were incubated with antibodies including CD29-PE (BioLegend, San Diego, USA), CD34-PE (R&D Systems, Minneapolis, USA), CD44-FITC (eBioscience, San Diego, USA), CD90-PE (BD Biosciences), CD45 (Abcam, Cambridge, UK) [44], CD73-PE (Bioss, Woburn, USA), CD105-FITC (Bioss), SSEA-1 (R&D Systems), SSEA-3 (R&D Systems), SSEA-4 (R&D Systems), TRA-1-60 (R&D Systems), and TRA-1-81 (Chemicon, Temecula, USA) [21] at room temperature for 1 h. The cells were washed twice with FACS buffer and resuspended in 500 μl of FACS buffer. The cells incubated with CD45, SSEA-1, SSEA-3, SSEA-4, TRA-1-60, or TRA-1-81 were incubated with anti-rat IgG, anti-rat IgM, anti-mouse IgG, and anti-mouse IgM secondary antibodies labeled with FITC for 1 h. Cells were then washed twice with FACS buffer and resuspended in 500 μl of FACS buffer. Cell fluorescence was evaluated by flow cytometry in a FACSCalibur instrument (BD Biosciences). Data were analyzed using Flowjo software (Tree Star, Ashland, USA).

### Reverse transcription and quantitative real-time PCR (qRT-PCR)

Total RNA was obtained from cultured BM-MSCs and AT-MSCs in passage 2. Total RNA was extracted using TRIzol reagent (Invitrogen) according to the manufacturer’s protocol. Total RNA was measured by spectrophotometry. Total RNA (1 μg) was reverse-transcribed at 42°C for 15 min in 20 μl with QuantiTect (Qiagen, Düsseldorf, Germany) after inactivation of reverse transcription by heating at 95°C for 3 min.

The cDNA product was subjected to real-time PCR according to the user instructions for the Real-Time PCR System 7300 (Applied Biosystems, Foster City, CA). qRT-PCR was performed at 95°C for 5 s and 60°C for 34 s in 20 μl buffer containing SYBR premix ExTaq II and ROX Reference Dye (Takara Bio, Shiga, Japan) and 0.2 μM each of the primers (Table2). Quantitative measurement was performed by establishing a linear amplification curve from serial dilutions of plasmid DNA containing each cDNA.

**Table 2:** Primers used for qRT-PCR

**Name**	**5’ – 3’**	**Direction**	**Position**	**Gen bank No.**
oct3/4-1	GCTCCTGAAGCAGAAGAGGA	sense	453–472	XM_538830
oct3/4-2	GCTGAACACCTTCCCAAAGA	anti-sense	540–559	XM_538830
sox2-1	CCCACCTACAGCATGTCCTA	sense	1177–1196	XM_545216
sox2-2	GGAGTGGGAGGAGGAGGTAA	anti-sense	1299–1318	XM_545216
nanog-1	CCCAACTCTAGGGACCCTTC	sense	22–41	XM_543828
nanog-2	CAGATCCATGGAGGAAGGAA	anti-sense	156–175	XM_543828
β-actin	GCCAACCGTGAGAAGATGACT	sense	339–360	AF021873
β-actin	CCCAGAGTCCATGACAATACCAG	anti-sense	446–468	AF021873

### Immunocytochemistry

Immunofluorescent staining was used to assess expression of the transcription factors Oct4, Sox2, and Nanog in BM- and AT-MSCs. Passage 2 cells were cultured in 4-well chamber slides (Nunc, Roskilde, Denmark) until 50% confluency. The cells were washed twice with PBS and fixed with 4% paraformaldehyde at room temperature for 30 min. After washing thrice with PBS, the cells were incubated with blocking solution containing 0.4% Triton X-100 and 4% Block Ace (DS Pharma Biomedical, Osaka, Japan) in PBS at room temperature for 1 h. The cells were incubated with rabbit polyclonal primary antibodies against Oct3/4 (Santa Cruz Biotechnology, Santa Cruz, USA), Sox2 (Stem Cell Technologies, Vancouver, Canada), and Nanog (Peprotech, Rocky Hill, USA) [21] diluted in blocking solution at 4°C for 16 h. The negative control cells were incubated without primary antibody and isotype control cells were incubated with normal rabbit IgG antibody (R&D Systems). The cells were washed thrice with PBS and incubated with secondary anti-rabbit antibody labeled with Alexa Fluor 488 (Invitrogen) diluted in blocking solution at room temperature for 1 h in darkness. The cells were then washed thrice with PBS and slides were mounted in Vectashield Hard Mounting Medium with DAPI (Vector Laboratories, Burlingame, USA). The cells were analyzed under a Zeiss Axiovert 200 M fluorescence microscope (Carl Zeiss MicroImaging, Jena, Germany), and image overlay was performed using Axio Vision Rel.4.6 software (Carl Zeiss MicroImaging) (Additional file 1: Table S1).

### Statistical analysis

Data were analyzed with an independent samples *t-test*, and a *P*-value of less than 0.01 was considered significant. Statistical analysis was performed using Prism software (GraphPad Software, San Diego, USA).

## Abbreviations

BM-MSC, Bone marrow-derived mesenchymal stem cell; AT-MSC, Adipose tissue-derived mesenchymal stem cell; ESC, Embryonic stem cell; qRT-PCR, Quantitative real-time polymerase chain reaction; SSEA, Stage specific embryonic antigen; TRA, Keratin surface-associated antigen tumor-related antigen.

## Competing interests

None of the authors has any financial or personal relationships that could inappropriately influence or bias the content of the paper.

## Authors’ contributions

HT and DZ designed the study, drafted the manuscript, and analyzed data; IY helped with editing and revision of the manuscript; YH helped with collection of samples and revision of the manuscript; MM helped with FACS analysis and revision of the manuscript; and TA contributed to the study design and helped with editing and revision of the manuscript. All authors read and approved the final manuscript.

## Supplementary Material

Additional file 1 **Table S1. ** Antibody Informations. Click here for file
