# Supplementary material for: Comparison of bone marrow and adipose tissue-derived canine mesenchymal stem cells
Source: BMC Vet Res. 2012 Aug 31;8:150. doi: 10.1186/1746-6148-8-150 (PMC3442961; doi:10.1186/1746-6148-8-150)
Supplement: Additional file 1 — Table S1. Antibody Informations. [file 1746-6148-8-150-S1.ppt]

## Slide 1
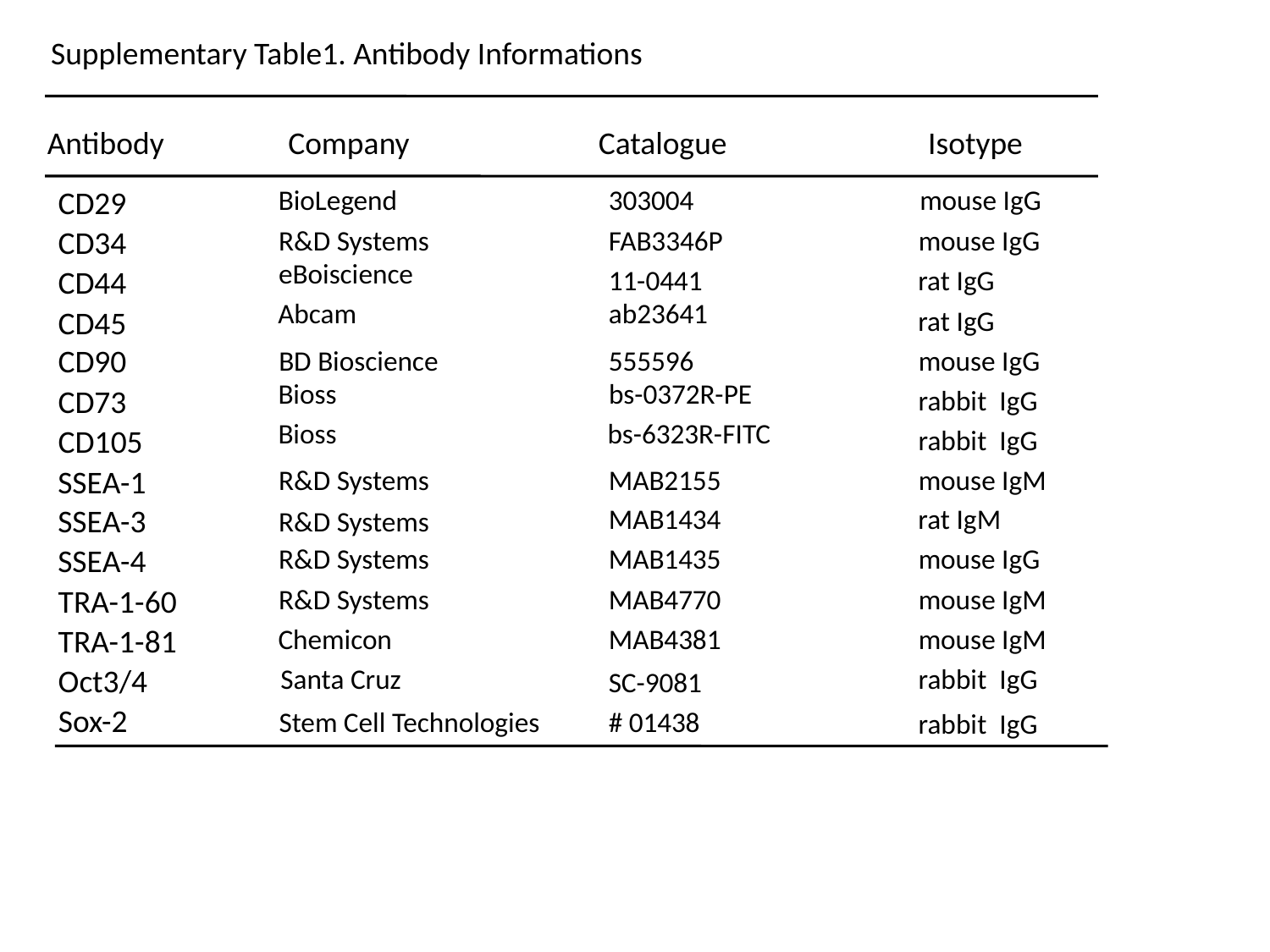

Supplementary Table1. Antibody Informations
Antibody
Company
Catalogue
Isotype
CD29
BioLegend
303004
mouse IgG
CD34
R&D Systems
FAB3346P
mouse IgG
eBoiscience
CD44
11-0441
rat IgG
Abcam
ab23641
CD45
rat IgG
CD90
BD Bioscience
555596
mouse IgG
Bioss
bs-0372R-PE
CD73
rabbit IgG
Bioss
bs-6323R-FITC
CD105
rabbit IgG
SSEA-1
R&D Systems
MAB2155
mouse IgM
SSEA-3
MAB1434
rat IgM
R&D Systems
SSEA-4
R&D Systems
MAB1435
mouse IgG
TRA-1-60
R&D Systems
MAB4770
mouse IgM
TRA-1-81
Chemicon
MAB4381
mouse IgM
Oct3/4
Santa Cruz
rabbit IgG
SC-9081
Sox-2
Stem Cell Technologies
# 01438
rabbit IgG
